# Animal Defenses against Infectious Agents: Is Damage Control More Important Than Pathogen Control?

**DOI:** 10.1371/journal.pbio.1000004

**Published:** 2008-12-23

**Authors:** Andrew F Read, Andrea L Graham, Lars Råberg

## Abstract

The ability of hosts to withstand a given number of pathogens is a critical component of health. Now playing catch-up with plant biologists, animal biologists are starting to formally separate this form of defense from classical resistance.

Once an infectious agent is established, hosts can do two things to minimize the agent's impact on their health. Most obviously, they can directly attack the growing pathogen population to contain or eliminate it. But hosts can also attempt to minimize the harm caused by a given number of pathogens, for instance by ramping up tissue repair and detoxifying pathogen by-products. “Resistance” and “tolerance,” as these two types of defense are known in the plant literature, were first distinguished by botanists in the late 1800s [1] and this distinction has been a focus of considerable work by plant scientists since then [2–4]. However, those advances have had a minimal effect on the study of animal diseases. Immunologists, microbiologists, and parasitologists have typically focused on the ability to limit parasite numbers (resistance) or the overall ability to maintain health irrespective of parasite burden (resistance plus tolerance), with little attempt to formally decompose human or animal health into resistance and tolerance components. That situation is only now beginning to change. The early results already have significant experimental and conceptual implications.

## Why Offense Is Not Always the Best Defense

One of the triumphs of 20th century immunology was the documentation in exquisite detail of the mechanisms animals have for killing infectious disease agents. Vaccination demonstrates that these mechanisms can be highly effective. Yet attack will not always work. Pathogens are frequently very slippery targets, with host–parasite coevolution generating a bewildering array of immuno-evasive or immuno-suppressive strategies. The organisms we recognize as pathogens are, by definition, staying ahead in host–pathogen arms races. This is partly because they generally have much shorter generation times than their hosts, but also because the fitness consequence of resistance is normally much more severe for pathogens than is the fitness consequence of infection for hosts. In antagonistic interactions, hosts are therefore doomed at best to ever-transient partial success. A second reason why attack is not always the best defense is that killing infectious agents can be very costly [5]. As in human warfare, attack can result in considerable self-inflicted collateral damage, and/or vast outlays in resources. Consequently, natural selection will, in many circumstances, favour the evolution of protective mechanisms that do not involve pathogen killing. It may even be that the majority of host defense mechanisms that have arisen during evolution are tolerance mechanisms. For one thing, natural selection is more likely to drive alleles conferring tolerance to fixation (reaching 100% frequency in a population) [6]. In contrast, resistance mechanisms work by eliminating parasites, and thus undermine the very selection pressures that favoured them in the first place. As a particular resistance mechanism nears fixation in a host population, parasites must change or die out, rendering the resistance mechanism unnecessary or useless. Tolerance will not prompt antagonistic counter-adaptation by pathogens, since it does not harm pathogen fitness [2]. Moreover, tolerance should have a neutral or even positive effect on pathogen prevalence. Hence, there will be continual selection in favour of a tolerance trait, even as it becomes common in the host population. Perhaps a very important evolutionary reason why pathogens do not make hosts even sicker is because an endless succession of tolerance mechanisms have gone to fixation through evolutionary time. The scientific focus on resistance may be because parasite killing mechanisms are both more easily observed and more likely to be genetically variable because of host–parasite coevolution. Now, however, experimentalists are beginning to turn their attention to damage control as well as pathogen control by animals.

## Evidence for Tolerance

**Figure 1:**
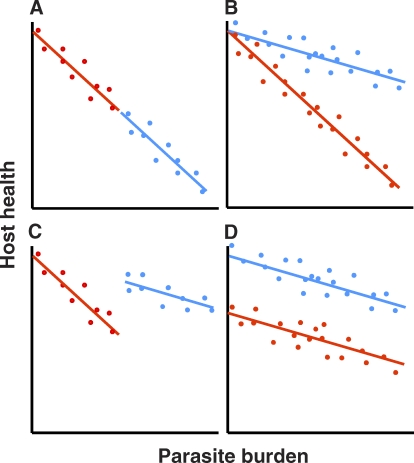
How to Statistically Separate Resistance and Tolerance Components of Host Health Dots represent individual hosts from one of two genotypes (red or blue) challenged with a fixed dose of a microparasite. (A) Both genotypes are similarly tolerant but differ in resistance, with the red genotype able to better reduce burdens and thus maintain a higher health status. (B) Host genotypes are equally resistant (similar mean burdens) but differ in tolerance, with red the less tolerant genotype because health declines more rapidly within increasing parasite burden. (C) Host genotypes differ in both tolerance and resistance; here, the more tolerant genotype (blue) is less resistant so that both genotypes have, on average, the same health status. (D) Host genotypes differ in neither resistance (they have the same mean burden) nor tolerance (they both have the same rate of decline in health as burdens increase). Their health differences arise from “general vigour,” because it is apparent even when no infection is present (intercept on the y-axis). This contrasts with the situation in (A), where the impact of increasing burdens on health is the same for both genotypes, and both are predicted to have the same health in the absence of infection. Reproduced from [9].

We believe the vast majority of scientists working on animal diseases would have little disagreement with the concept of tolerance (although they may disagree with the semantics; see Box 1). This is because a considerable body of data is anecdotally consistent with tolerance in animals [7]. For instance, unlike sickle cell anaemia in humans, which is a classical resistance factor because it reduces malaria parasite densities, α^+^-thalassaemia (another heritable blood disorder) is very likely a tolerance factor. It does not affect malaria parasite densities, but is nonetheless associated with reduced incidence of “severe” (life-threatening) malaria [8]. But to generate a science of tolerance, the phenotype needs to be clearly measurable, independently of resistance. It is difficult yet to say whether α^+^-thalassaemia is definitely a tolerance factor, because there are several factors that may generate spurious variation for tolerance. Determining the roles of resistance and tolerance in health outcomes requires their statistical separation, which, for a variety of reasonably tedious statistical reasons [7], is best done using the approach developed in plant biology (Figure 1). This requires measuring health outcome and parasite burdens and then studying that component of variation in health outcome which is not attributable to resistance (pathogen numbers). Attempts to do this systematically for animal disease are only just beginning.

**Box 1. Words about Words**

We are only too aware that some readers will have been irritated by the way we use the terms “resistance” and “tolerance” (and perhaps even “defense”) in this Primer. The difficulty is that the separate traditions, educational cultures, and independent historical development of the many disciplines involved in host–parasite studies have led to semantic chaos. Worse, microbiologists, immunologists, ecologists, parasitologists, vets, and physicians often adhere vehemently to their particular definitions.

Many in the biomedical community use “tolerance” to mean things other than what we and the plant science community mean. For example, different breeds of cattle in Africa are often classified as being tolerant or not to infection by trypanosomes, but here tolerance describes the overall impact of infection on disease severity and host fitness, irrespective of parasite burden [17]. Many immunologists describe immunological non-responsiveness as “tolerance” [18,19]. Yet a different definition is the ability to avoid immunopathology [20]. Given that “tolerance” already has divergent definitions in biomedicine, none of which align with our definition, perhaps we should use another word to describe the ability to withstand a given parasite burden? We do not, partly because the obvious alternatives (e.g., resilience, endurance) also already have alternative meanings in biology. More importantly, though, the plant science definition of tolerance has a long pedigree, and it is now very precisely and quantitatively defined as the slope of a regression of health or fitness against parasite burden (Figure 1).

We note that because tolerance already means a variety of things to different animal disease biologists, explicit definitions are always needed, and with those, any ambiguity is banished. Our definition of tolerance is the ability to limit the health or fitness consequences of a given parasite burden, whereas resistance limits parasite burdens (“burden” is itself defined with a system- and discipline-appropriate measure such as pathogen/parasite titre, density, or biomass). Tolerance and resistance are thus two different but complementary host traits that together determine how harmful an infection is. A crucial difference between these two components of host defense is that resistance has a negative effect on the performance of the parasite, whereas tolerance does not; as discussed in the main text, this difference has important ecological and evolutionary implications.

For example, we recently dissected resistance and tolerance to rodent malaria in laboratory mice [9]. Using anaemia and weight loss as measures of the effects of infection on host health, and measures of parasite densities to estimate host resistance, we were able to formally show that in addition to the well-known genetic variation in resistance to malaria, there is also genetic variation in tolerance. Interestingly, although we worked with only five mouse strains, there was nonetheless a perfect negative relationship between tolerance and resistance: those mouse strains that most controlled their parasite burdens (i.e., high resistance) were those whose health was most affected by small changes in parasite burden (i.e., low tolerance).

## Mechanisms and Genes

Elucidation of the mechanistic and genetic basis of tolerance traits is of interest in its own right, not least in this age of extreme reductionism, when many in biomedicine simply do not believe something exists until the molecular pathways involved have been revealed. Mechanisms of tolerance are likely to include increased investment in vulnerable tissues, both before and after attack. Hosts with thicker gut linings will be less affected by grazing nematodes, and those able to more rapidly replace red cells will be less affected by severe malaria anaemia. Immunological mechanisms will also be involved. Tolerance may involve “anti-disease immunity” or “anti-toxin immunity,” where responses are not directed at the parasite itself, but rather at toxins and other harmful substances produced by the parasite. Tolerance may also involve mechanisms which damp down inappropriate host responses and/or limit collateral damage (“immunopathology”) from otherwise well-directed immune responses [7]. Importantly, a particular mechanism may affect both resistance and tolerance. Thus, tolerance will almost certainly involve the great variety of mechanisms involved in resistance and many more besides. Yet it is striking how little we know about tolerance mechanisms relative to resistance mechanisms—and indeed that the tolerance mechanisms we understand best are those that mitigate the side effects of resistance.

When it comes to genes, the situation is even worse. Even in plants, where the existence of genetic variation for tolerance has been long known, genes responsible for this anti-disease variation have not yet been identified at a molecular level. Similarly, the mouse genes responsible for our observations [9] have yet to be identified. Elucidating genes involved in tolerance will be important for several reasons. First, it is a way to determine what types of mechanisms are involved and their relative importance (tissue repair? immunological?). And if we are lucky, it may be possible to infer from the genetic basis something about underlying genetic relationships between resistance and tolerance, and the evolutionary processes involved in maintaining genetic variation in both.

New research by Ayres and Schneider published in this issue of *PLoS Biology* [10] reveals all this and more—and all for a single mutation. Earlier this year, the same authors [11] reported a screen of 1,000 mutant fruit fly lines from which they identified 18 more likely to die from infection with the intracellular bacterial pathogen Listeria monocytogenes. Of the 18, infection intensities were higher in 12, suggesting that these were resistance-defective mutants. The remaining six, though, died faster without elevated pathogen titres, and so were most likely tolerance-defective mutants. The phenotypic description of these mutants came just two years after the existence of genetic variation in tolerance in flies was first proposed [12] and 114 years after tolerance was recognised by plant scientists.

Ayres and Schneider's latest work [10] is an analysis of the resistance and tolerance phenotypes of flies with a single mutation in *CG3066*, a gene encoding a protease active in the melanisation cascade, an innate immunity pathway in invertebrates. By challenging wild-type and CG3066-defective flies with one of a panel of seven bacterial species, they revealed that this protease can be viewed as a tolerance gene, a resistance gene, or neither, depending on the pathogen involved. Thus, with some bacterial species, the mutant flies were resistance-defective, dying faster of overwhelming bacteria. With other species, the same mutation enhanced resistance, with the flies harbouring fewer pathogens and being less likely to die. With two other bacterial species, fly lifespan was unaffected, but for one, the mutant line had lower burdens (resistance-enhanced, tolerance-defective), and for the other, the mutant line had more bacteria (resistance-defective, tolerance-enhanced). In an earlier study [13,14], these same authors reported that mutants in the fly Tumour Necrosis Factor–related molecule “eiger,” when challenged with one bacterial species, lived longer with similar pathogen titres (tolerance-enhanced), and yet when challenged with another species, died faster with reduced titres (resistance-enhanced, tolerance-defective). Thus, ironically, the first evidence of tolerance genes in flies shows that it does not make sense to talk of “resistance” or “tolerance” genes at all: one gene can be involved in tolerance and resistance, depending on the pathogen.

## Implications

On a practical level, this work has a depressing conclusion: more work is required, even to verify what we think we know now about fly immunity. It is clearly critical to measure infection burdens, not just health outcomes, so that it is possible to analyse both the intensity-dependent health outcome, and variance in health outcome that is over and above that due to infection intensities. Worse, it is not possible to assume that a particular gene is uninvolved in defense based on experiments with a single pathogen, or even a limited number. A panel of pathogens, and hence much larger experiments, are required.

More positively, the finding that a single mutant can be associated with increased or decreased resistance and increased or decreased tolerance immediately suggests a mechanism by which genetic variation in resistance and tolerance can be maintained in nature. As described above, simple theory predicts that tolerance traits will go to fixation in a population [6]. Yet hosts naturally face a highly diverse set of pathogens. If Ayres and Schneider's findings [10] generalise, so that high tolerance to one pathogen is associated with low tolerance to another—or that resistance and tolerance are negatively genetically correlated—then diverse pathogen faunas will generate diverse selection pressures on defense mechanisms, so that nothing can go to fixation. Load on top of that resistance alleles in a host population which will change the exposure to host-specific pathogens, and there is outstanding potential for marked population and evolutionary dynamics. This would be further enhanced if host genetic background is also important (yet to be tested). All this mind-boggling complexity does have one positive practical note: if genetic variation in tolerance is readily maintained by these mechanisms, it should be easy to find and elucidate tolerance traits, analyse their implications for host health and pathogen epidemiology, and determine the factors driving tolerance evolution.

## Prediction and Application

Unlike sciences such as physics and evolutionary biology, immunology has not to date been much motivated by predictive synthetic theories. Now that it is clearly possible to empirically partition defenses into tolerance and resistance, it should be possible to develop and test hypotheses about their relative importance in different circumstances. A natural hypothesis is that the fitness benefits and costs of resistance and tolerance vary across environmental conditions, favouring different combinations of these two components of defense under different circumstances [15]. For example, it has been argued that a high rate of infection but low virulence should select for host tolerance, whereas the opposite should favour resistance [16]. Similar adaptive scenarios can be envisaged for variation in, for example, host reproductive status and age and body condition. Testing such adaptive hypotheses would move the study of animal defenses beyond elucidation of mechanism.

The existence of variation in the ability to withstand a given pathogen burden is also of more than academic interest. As plant scientists have argued [2], artificial increases in tolerance by selective breeding may be more evolution-proof than manipulations in resistance, because tolerance does not impose selection for pathogen countermeasures. By analogy, public or animal health interventions that increase tolerance may be less likely to fail in the face of pathogen evolution than are interventions that increase resistance. In agricultural animals, attempts to select for increased yield in the face of parasite challenge may come to nothing (or even make things worse) if there is a trade-off between resistance and tolerance. Thus, explicit analyses of the tolerance component of host defense are bound to be useful – and interesting. Not least, we should soon know whether tolerance is as important as resistance in determining the fate of infected animals.
